# The Medical Profession—A Contributory Factor to the Death Rate of Consumptives

**Published:** 1904-08

**Authors:** C. H. Wilkinson

**Affiliations:** San Antonio, Texas


### TEXAS MEDICAL JOURNAL.

ESTABLISHED JULY, 1885.

PUBLISHED MONTHLY.—SUBSCRIPTION $1.00 A YEAR.

VOL. XX. AUSTIN, AUGUST, 1904. No. 2.

### Original Contributions.

For Texas Medical Journal.

### The Medical Profession—A Contributory Factor to the Death Rate of Consumptives.

BY C. H. WILKINSON, M. D., SAN ANTONIO, TEXAS.

At the first glance the proposition just stated would seem to be a libel on a profession which, from time immemorial, has been recognized as the conservator of health and a bulwark between humanity and death. And right truly can this be asserted as regards nearly every malady on earth except in the case of the one under consideration. There is no body of men more charitable to humanity than ours. There is no calling more self-sacrificing and more zealous in its efforts to save human life than that of medicine.

Through the patience and untiring energy of physicians, great epidemic diseases have been brought under subjection, and the death rate amongst individuals has been reduced through their efforts at least 20 per cent in the past fifty years.

They have raised the profession to a point of dignity and honor, and their successes are in keeping with those of any science known. But there is a reverse to this picture, and across its face should be written, in dark characters, the word consumption.

For centuries past this great devastator of the human race has engaged the attention of medical men. At sea—in the beginning —as to its etiology, they simply did what they considered best for its relief, and to their efforts be praise and commendation forever. By degree, however, the mists that obscured our subject have gradually been wafted away, so that today there is no excuse for ignorance as to the nature, cause and diagnosis of this disease.

Every doctor now knows, or should know, the cause of consumption, and every physician should know the usual termination of such cases under the ordinary futile course pursued for their relief. There is no excuse for delaying an accurate diagnosis any longer. The microscope reveals its nature in a few minutes in the majority of instances, and then it is up to the physician to assert his manhood by revealing the nature of the malady to his confiding patients, when he meets them. Unfortunately, however, the lack of this professional manhood too frequently prevails, and we withhold that candor which we in honor owe to those whose lives depend upon it.

Through fear of "shock" to his feelings, we evade the inquiry so often made by the victim, "Doctor, have I got consumption?" In this evasion we contribute to his death. In this evasion we rob him of time, precaution and opportunity, each in itself invaluable in the management of such cases.

We thereby deceive our patrons into the delusion that they have not phthisis, but, nevertheless, we place them on consumptive treatment and ask them to call again at our office for further consultation. We are perfectly familiar with the facts that the usual routine treatment of consumptives with drugs generally ends in death, and that all experimentation with fads and systems usually terminates the same way.

We know that in climatic change, in the early stage of the disease, and in the open air life in some salubrious atmosphere, we can expect more benefit than in any other agencies known to the profession.

We know that incipient cases, mainly, are amenable to cure, and that they can reasonably expect it in selected climate.

All these things we know, and yet we continue to handle our cases from our pharmacopeas, at a dollar an office call, until the advanced stage of phthisis has arrived, and the patient is beyond the reach of climate or of any curative agency.

Again, having dilly-dallied with our phthisical cases until the hopeless third stage has arrived, we tell them to "go west," get out into Arizona or some other remote territory, where they, unconsigned to any particular place or person, and wasted in flesh and finances that might have been applied to an earlier pilgrimage, soon die of ennui and tuberculosis combined.

Thus we err in two particulars in the management of our cases. Instead of sending them away in the early stage of their disease to a climate suited to their condition—which we ought to do—we temporize with them until the opportunity for relief is passed, and then, instead of keeping them at home to die amongst their friends and kindred, we send them off to die, "a stranger in a strange land."

The writer has been in charge of a camp for consumptives for the past two years, and has had abundant opportunities for verifying the statement made above.

Kept at home until the last stage of their disease had been reached, many of these poor victims realized too late that they had not been treated candidly at home.

"Had my physician at home only told me in the beginning that I had consumption, I would have been here long ago, and maybe well today." This and similar expressions have been heard so often that they have acted as an incentive to the writing of this article.

In this connection, I would suggest that, in the manner of handling consumptive cases at their homes, the medical profession might be divided into the following classes:

First. Those physicians who promptly inform their patients of the nature of their disease, and insist upon their seeking suitable climate for their relief at once.

Second. Those who withhold such advice, and allow their cases to remain at home, under the belief that they can be relieved by some new or attractive medical treatment.

Third. Those who, through lack of moral courage, neglect to state their patient's disease, for fear of shocking their tender sensibilities, until its nature is apparent to all beholders.

Fourth. Those, who knowing well the result of procrastination in such cases, retain and treat them at home for the money they can make out of them.

All but class number one are contributory factors in the death rate of consumptives.

### OUR DUTY.

In handling cases of tuberculosis, a physician should bear in mind two important facts. First, he should know that nearly all such cases die unless they make at once a radical change of climate from the one they contracted their disease in; and, secondly, it is not only useless, but *actually* inhuman to ship them off from home and into a land of strangers after they attain the hopeless stage.

As soon, therefore, as we suspect a tubercular nature in his symptoms, we should urge him to go at once to a climate known to be renowned for its effects upon such cases, and there are scores of them well known to the profession in many of our Western and Southwestern States.

In his early stage he can shift for himself, after he completes his journey; in the last stage of phthisis he can not.

In the early stage, before grave molecular changes have taken place in the lungs, the bacilli will almost always quickly die out under the sunshine, pure air and other oxidizing influences of his new abode in a suitable climate; but when once such lesions have occurred recovery is rare; and, when systemic infection to much extent has taken place, then climate is of no avail, and all such cases should be kept at home, to run their usual course.

### CONSIGNING CASES.

Apropos to the subject under consideration, every physician contemplating sending pulmonary cases away from home for climatic or for any other treatment, should first secure some reliable person at the place of destination to whom such cases should be consigned. This is an important matter and one that should never be neglected if possible by the medical adviser. Such consignee need not be a physician, though it were preferable that he should. Almost any honest and experienced layman might suffice in the absence of a good physician, but for many and obvious reasons the latter is preferred.

The majority of phthisical patients arrive at their destination complete strangers to all their new surroundings. They know no one, as a rule, neither do they know how or where to select their abodes, nor the life to lead. The result of this ignorance, in many instances, is a landing made in some illy-suited, cheap and unsanitary boarding house, where they reinfect themselves by inheriting disease germs left by some late decedent, or else they fall into the hands of some professional fake more dangerous in reality than the bacilli themselves.

Select you some respectable physician at the other end of the line, if possible, and formally consign your patient o him. The medical consignee will thereby take more than the usual professional interest in the invalid, and see to it that his personal, as well as medical, wants are not neglected, as they usually otherwise are.

The health seeker, on the other hand, feels that he has then an acquaintance and a friend in his new physician, and thus one of the most disagreeable, if not one of the most depressing agencies on a consumptive—lonesomeness—is largely averted.

Before closing these lines, and for the information of physicians having tubercular cases under treatment, I would call brief attention to the climate of West Texas for the relief of such. When sent to this region in their incipiency, it is no exaggeration to state that no finer climate can be found for them in all America.

Especially do these remarks apply to the country west and northwest of San Antonio. Here the atmosphere is dry and salubrious, and pyogenic germs can not thrive unless carefully nurtured, and the bacilli of tuberculosis soon die out in the presence of its magnificent influence. Here hundreds of health seekers annually resort in order to recuperate, and find cure and benefit by remaining in the open air for the period of a few months.

This country is easily reached by rail, three large lines running through it out of San Antonio, while the requirements of the consumptives can be obtained either in hotels, camps or on ranches.

And now, with this information furnished the physician, there is no reason for his not knowing where to send his patient, and in the modern and accessible lights to diagnosis afforded them, there is no longer any excuse for procrastination in securing them relief.

